# Age Bias in Zebrafish Models of Epilepsy: What Can We Learn From Old Fish?

**DOI:** 10.3389/fcell.2020.573303

**Published:** 2020-09-10

**Authors:** Sung-Joon Cho, Eugene Park, Andrew Baker, Aylin Y. Reid

**Affiliations:** ^1^Division of Fundamental Neurobiology, Krembil Research Institute, University Health Network, Toronto, ON, Canada; ^2^Collaborative Program in Neuroscience, University of Toronto, Toronto, ON, Canada; ^3^Keenan Research Center, St. Michael’s Hospital, Li Ka Shing Knowledge Institute, Toronto, ON, Canada; ^4^Department of Anesthesia and Surgery, University of Toronto, Toronto, ON, Canada; ^5^Department of Medicine, University of Toronto, Toronto, ON, Canada

**Keywords:** zebrafish, adult, larva, epilepsy, seizure, blood-brain barrier, neurogenesis

## Abstract

Zebrafish are a powerful tool for investigating epilepsy. Mammalian seizures can be recapitulated molecularly, behaviorally, and electrophysiologically, using a fraction of the resources required for experiments in mammals. Larval zebrafish offer exceptionally economical and high-throughput approaches and are amenable to state-of-the-art genetic engineering techniques, providing valuable transgenic models of human diseases. For these reasons, larvae tend to be chosen for studying epilepsy, but the value of adult zebrafish may be underappreciated. Zebrafish exhibit transient larval – adult duality. The incompletely developed neural system of larval zebrafish may limit the translation of complex neurological disorders. Larval zebrafish go through dynamic changes during ontogenesis, whereas adult zebrafish are physiologically more stable. Adult zebrafish have a full range of complex brain structures and functions, such as an endothelial blood-brain barrier and adult neurogenesis, both are significant factors in epilepsy research. This review highlights the differences between larval and adult zebrafish that should be considered in pathophysiological and pharmacological studies of epilepsy.

## Introduction

Epilepsy is a common neurological disorder, affecting over 70 million people worldwide (Thijs et al., 2019). An imbalance of excitatory and inhibitory processes causes unprovoked seizures and other behavioral changes despite optimal treatment (McCormick and Contreras, 2001). In addition to seizures, people with epilepsy frequently experience cognitive and psychological impairment, pervasive social stigma, and early death (McCormick and Contreras, 2001; Thijs et al., 2019).

There are three primary treatment modalities for epilepsy: anti-seizure drugs (ASDs), resective or palliative surgeries, and neurostimulation. The first-line approach is ASDs. However, this treatment is only effective in treating seizures and not the underlying epileptic disorder. Moreover, ASDs often cause adverse effects, and a third of people with epilepsy develop drug resistance (Thijs et al., 2019). Surgical removal or disconnection of an epileptic brain region can be more effective for drug-resistant focal epilepsy, but only a small number of patients are eligible for surgery (Ryvlin et al., 2014). For those who are ineligible, neuromodulation is increasingly used, but rarely results in seizure-free status (Kwon et al., 2018). Therefore, despite this field’s long history, innovative approaches to prevent or disrupt epileptogenesis are still needed. For this purpose, a good experimental model is essential.

The ideal animal model of epilepsy should reflect the processes underlying the disorder in humans including electrophysiological, neuroanatomical, biochemical, and genetic factors. Rodents are often the default experimental models for epilepsy as they are for many other conditions, but it must be kept in mind that all animal models are comparative. The model chosen must be the most appropriate for addressing a particular research question, and the strengths and limitations of epilepsy models lie in how they recapitulate human epilepsy (Grone and Baraban, 2015). Models in animals other than rodents are becoming increasingly important as they possess complementary advantages to studying epileptogenesis and evaluating treatments.

Zebrafish first started to receive attention as a valid animal for studying epilepsy in 2005, when it was demonstrated that they could express chemically induced seizure phenotypes that would be expected from rodents (Baraban et al., 2005). Since then, the zebrafish has established its role as a desirable alternative to rodents. Zebrafish are vertebrates with a fully sequenced genome and significant genetic homology with humans: 84% of human disease-associated genes are expressed in zebrafish (Howe et al., 2013). The zebrafish brain is also remarkably similar to rodents in terms of macro-organization and cellular morphology (Kalueff et al., 2014). Furthermore, the overall time and cost of studies using zebrafish are lower, the animals are well suited to high-throughput screening, and they have a lifespan of 5 years, at least 50% longer than that of commonly used mouse strains (Gerhard and Cheng, 2002). Maintenance costs of zebrafish are 1000 times less than the cost of rodents in similar studies (Ahmed et al., 2014), and drug screens are estimated to be 500 times cheaper than similar rodent assays (Steenbergen et al., 2011).

Zebrafish are considered adults from 90 days post-fertilization (dpf), and are sexually active until death. They breed year-round, and a single female can lay hundreds of eggs a week. Zebrafish embryos grow externally, and the larvae are highly transparent until 2–3 weeks of development, allowing exceptionally detailed visualization of their embryogenesis and central nervous system. Due to the easy optical access, the larvae are particularly well suited to the application of calcium imaging and optogenetic approaches in epilepsy studies (Turrini et al., 2017; Diaz Verdugo et al., 2019). Moreover, embryonic zebrafish are amenable to traditional and modern genome engineering approaches in an easier, cheaper, and faster manner, such as those using morpholinos, RNA interference, synthetic capped mRNA injection, expression plasmid injection, zinc finger nucleases (ZFNs), transcription activator-like effector nucleases (TALENs), and clustered regularly interspaced short palindromic repeats (CRISPR) (Koster and Sassen, 2015). The availability of a state-of-the-art genome engineering toolbox in zebrafish larvae has enabled the study of genetic aspects of epilepsy in a high–throughput manner and has been exploited for personalized drug discovery, by offering epileptic mutation models such as Batten disease (Wager et al., 2016), and Dravet syndrome (Baraban et al., 2013).

Zebrafish do not require feeding until 4-dpf, and they absorb drug compounds directly from their bathing medium. Larvae can, therefore, be kept in multi-well plates, and their behavioral symptoms can be monitored by video-tracking devices. Automated pipetting, sorting, drug delivery, and medium changes have been employed in multi-well setups, aiding high–throughput screening. For these reasons, young larvae (younger than 7-dpf; Table 1) tend to be chosen for zebrafish studies, but the benefits of adult zebrafish are often underestimated. Larval studies often rely on genetic epilepsy models and usually lead to early death. For example, *cln3* mutants only live up to 6-dpf (Wager et al., 2016) and *scn1lab* mutants can survive up to 13-dpf (Eimon et al., 2018), therefore findings from these models are focused on a very small time window in the zebrafish life span. In addition, the incompletely developed neural system in larvae may limit the investigable aspects of epilepsy. While larval zebrafish exhibit dynamic changes through their ontogenesis, adult zebrafish are physiologically more stable. Adult zebrafish have a full range of complex brain functions with higher behavior and integrated neural functions including memory, conditioned responses, and social behaviors (Santana et al., 2012). Such differences between larvae and adults can lead to differences in drug screening results (Table 2). In this review, we highlight factors to consider when selecting zebrafish of an appropriate age for epilepsy studies.

**TABLE 1:** Examples of epilepsy studies using zebrafish (2016–2020).

**Age**	**Seizure type**	**Mutant**	**Phenotyping paradigm**	**Study references**
4-dpf	Batten disease	CLN3	LFP	Wager et al., 2016
7-dpf	Febrile	STX1B	LFP	Hunyadi et al., 2017
Approx. 7-dpf	Dravet syndrome	SCN1A	LFP	Eimon et al., 2018
Approx. 8-dpf	PTZ	Wild-type	Calcium imaging	Diaz Verdugo et al., 2019
Approx. 8-dpf	PTZ	Wild-type	LFP	Hunyadi et al., 2017
Adult	Pilocarpine	Wild-type	Behavioral assay	Paudel et al., 2020
Adult	Caffeine	Wild-type	Behavioral assay	Vada et al., 2017
Adult	PTZ	Wild-type	Behavioral assay	Choo et al., 2019
Adult	PTZ	Wild-type	Multichannel EEG	Cho et al., 2017
Adult	Post-traumatic	Wild-type	LFP	Ref

*dpf, days post-fertilization; PTZ, pentylenetetrazol; LFP, single-channel local field potential; EEG, electroencephalography.*

**TABLE 2:** Antiepileptic drugs that have been tested in zebrafish.

**ASDs**	**Larvae**	**Adults**	**Study references**
**Focal/generalized seizures**			
Benzodiazepines	+ PTZ, DS* − EAST^†^	+ PTZ	Berghmans et al., 2007; Baraban et al., 2013; Mussulini et al., 2013; Zdebik et al., 2013; Gupta et al., 2014; Kundap et al., 2017
Lamotrigine	+ PTZ − DS*	+ PTZ	Berghmans et al., 2007; Afrikanova et al., 2013; Baraban et al., 2013; Martinez et al., 2018; Choo et al., 2019
Levetiracetam	− AG, DS*	+ PTZ	Afrikanova et al., 2013; Baraban et al., 2013; Leclercq et al., 2015; Martinez et al., 2018; Choo et al., 2019
Perampanel	n/a	+ PTZ	Choo et al., 2019
Phenobarbital	− PTZ	n/a	Martinez et al., 2018
Topiramate	+ PTZ, AG	n/a	Berghmans et al., 2007; Afrikanova et al., 2013; Leclercq et al., 2015
Sodium valproate	+ PTZ, AG, DS*	+ PTZ	Berghmans et al., 2007; Afrikanova et al., 2013; Baraban et al., 2013; Gupta et al., 2014; Leclercq et al., 2015; Martinez et al., 2018; Choo et al., 2019
Zonisamide	+ PTZ	+ PTZ	Berghmans et al., 2007; Choo et al., 2019
**Focal seizures**			
Brivaracetam	n/a	n/a	
Carbamazepine	+ DS* − PTZ	+ PTZ	Afrikanova et al., 2013; Baraban et al., 2013; Gupta et al., 2014; Martinez et al., 2018
Eslicarbazepine acetate	n/a	n/a	
Gabapentin	− PTZ	+ PTZ	Afrikanova et al., 2013; Banote et al., 2013; Gupta et al., 2014; Kundap et al., 2017
Lacosamide	n/a	+ PTZ	Gupta et al., 2014
Oxcarbazepine	− PTZ	+ PTZ	Afrikanova et al., 2013; Kundap et al., 2017
Phenytoin	+ AG, PTZ − PTZ	+ PTZ	Afrikanova et al., 2013; Leclercq et al., 2015; Kundap et al., 2017; Martinez et al., 2018
Pregabalin	n/a	− PTZ	Gupta et al., 2014
Tiagabine	+ PTZ	n/a	Berghmans et al., 2007; Afrikanova et al., 2013
Vigabatrin	n/a	n/a	
**Absence seizures**			
Ethosuximide	+ PTZ − DS*	n/a	Berghmans et al., 2007; Afrikanova et al., 2013; Baraban et al., 2013
**Special encephalopathies**			
Cannabidiol	n/a	n/a	
Everolimus	n/a	n/a	
Felbamate	n/a	n/a	
Rufinamide	n/a	n/a	
Stiripentol	+ DS*	n/a	Baraban et al., 2013

List of AEDs adapted from Thijs et al. (2019) PTZ, pentylenetetrazol; DS, Dravet syndrome; AG, allylglycine. *SCN1A mutants; ^†^KCNJ10 mutants; n/a, not assessed.

## Morphological Changes From Larva to Adult

In a typical laboratory setting, zebrafish go through up to 6 weeks of larval development. During this stage, they grow more than three times in length and exhibit morphological changes, such as muscle structure development, fin transformation, and adult pigment patternings (Thorsen and Hale, 2005). Juvenile zebrafish (<90-dpf) lose the larval fin fold and acquire scales (Singleman and Holtzman, 2014), and endoskeletal elements are formed and connected to the cranial area (Thorsen and Hale, 2005). Just like other teleosts, zebrafish undergo remodeling of organs, the sensory system, and the central and peripheral nervous systems during maturation (McMenamin and Parichy, 2013). Juvenile zebrafish are also emerging in the field of neuroscience as valid models in learning (Valente et al., 2012) and social behaviors (Dreosti et al., 2015). Also, the availability of techniques to image brain activity in juvenile zebrafish (Jetti et al., 2014) could make them a useful model organism to study epilepsy. While there is a scarcity of seizure studies in zebrafish at this age, a genetic model of juvenile myoclonic epilepsy has demonstrated convulsive seizures upon light exposure in juvenile zebrafish (Samarut et al., 2018).

Zebrafish are considered adults after acquiring all their adult organs and becoming sexually mature, which typically happens after 90-dpf (Singleman and Holtzman, 2014). Stable physiology in adulthood is particularly advantageous in longer-term studies. It allows the evaluation of long-term therapeutic effects, and the generation of chronic seizure states. Until recently, the generation of a chronic epilepsy-like condition outside of genetic manipulation in zebrafish had been unachieved. Recurrent administration of pilocarpine (Paudel et al., 2020) or pentylenetetrazole (PTZ) (Duy et al., 2017) does not induce recurrent spontaneous seizures. However, we recently modeled post-traumatic epilepsy (Cho et al., 2020b), which is the first adult zebrafish model that exhibits spontaneous seizures. This model also demonstrates disruption of the blood-brain barrier, increased markers of inflammation, and alterations in tau phosphorylation mTOR signaling, mechanisms relevant to epileptogenesis that cannot be as easily studied in larvae.

## Sex Differences in Zebrafish

Although the role of sex in epilepsy is not clear, it is a critical biological variable that should always be considered in both clinical and preclinical settings. Sex hormones are known to play a role in seizure susceptibility and the development of epilepsy in both rodents and humans (Mejías-Aponte et al., 2002). Sex differences not only influence epilepsy itself, but also affect the metabolism of ASDs (Scharfman and MacLusky, 2014).

Visual sex identification of zebrafish is only possible in adulthood. There are sex differences in zebrafish gene expression, including neural genes that are similar to those in humans and rodents (Wong et al., 2014). Other sex differences in the brain include a higher rate of cell proliferation in the cerebellum of male zebrafish (Ampatzis and Dermon, 2007), and higher hypothalamic and cerebellar metabolic activities in females (Ampatzis and Dermon, 2016). An increasing number of neuropsychological zebrafish studies have identified sex differences in aggression, social preferences, anxiety, and learning/memory, including differences in behavioral changes after cocaine exposure (López Patiño et al., 2008). However, sex differences are routinely ignored in zebrafish epilepsy studies – even those using adults. To date, only a few studies examined sex differences (and found none) in behavioral and electrophysiological seizure development using PTZ in zebrafish (Braida et al., 2012; Cho et al., 2017). The lack of interest in sex differences may lead to an incomplete understanding of disease processes and treatment responses. The use of adult zebrafish may provide a valuable contribution to understanding the influence of sex in epilepsy and its treatments, something that cannot be easily done in larval zebrafish studies.

## The Blood-Brain Barrier (BBB) and Epilepsy

The BBB is a physical and metabolic interface that regulates and protects the brain’s microenvironment by limiting the penetration of external substances into the brain (Oby and Janigro, 2006). The mammalian BBB is mainly formed from endothelial cells associating with pericytes and astrocytes and functions as a permeability barrier (Jeong et al., 2008). BBB disruption is associated with various neurological diseases, including the cause, effect, and treatment of seizures (Oby and Janigro, 2006). BBB disruption may lead to epileptic seizures and, conversely, acute seizures can cause breakdown of the BBB with subsequent development of epilepsy. The level of BBB permeability positively correlates to seizure frequency in a temporal lobe epilepsy model (Van Vliet et al., 2007) and artificial opening of the BBB can generate epileptogenic foci (Seiffert et al., 2004; Van Vliet et al., 2007) supporting the BBB as a possible target to prevent epileptogenesis. The presence of the BBB is also a bottleneck in the development of epilepsy medications, as the tight barrier can cause significant problems in delivering ASDs to the brain. A better understanding of the role of the BBB is imperative in epilepsy research.

The functional and structural complexity of the zebrafish BBB is similar to that of higher vertebrates. It starts to form at 3-dpf, when expression of zonula occludens-1 (ZO-1) [tight junction protein-1 (TJP-1)] and claudin-5 is observed, and is fully formed and functional at 14-dpf (Jeong et al., 2008; Fleming et al., 2013). The function of the BBB also matures with its structural development. Immature BBB structures are more permeable than adult structures, with sulf-NHS-biotin (443-Da) and Evans blue (961-Da) traversing the BBB at 3-dpf (Jeong et al., 2008; Fleming et al., 2013), and sodium fluorescein (376-Da) traversing it at 8-dpf (Fleming et al., 2013). Adult zebrafish possess an endothelial cell-based BBB that is functionally and molecularly comparable to that in mammals and contains tight junctions between overlapping brain endothelial cells (Jeong et al., 2008), astrocytes and the basal lamina (Brian et al., 2011). Tight junctions are the key structures of BBB, responsible for size-dependent exclusion of molecules (Wolburg et al., 2009). As the ultrastructure and function of the zebrafish BBB matures during development, it should not be assumed that a similar role of the BBB in epileptogenesis and the therapeutic effects of drugs would be observed in embryonic and adult zebrafish.

## Adult Neurogenesis

Neurogenesis is a phenomenon that generates new mature, functional neurons in the brain. The role of seizure-induced neurogenesis in epileptogenesis remains unclear, but new insights have been gained in cellular remodeling after seizure events. We know from rodent models that acute seizures elevate adult neurogenesis in the subgranular zone (SGZ) and the subventricular zone (SVZ) (Parent et al., 1997, 2002; Marta et al., 2011). Most rodent epilepsy models show dramatically altered neurogenesis with two significant features: formation of hilar basal dendrites and ectopic migration of newborn neurons (Jessberger and Parent, 2015). Seizure-induced neurogenesis and the resulting aberrant neuronal connections are recognized to promote the process of epileptogenesis (Duy et al., 2017; Neuberger et al., 2017). Suppression of neurogenesis also reduces epileptogenesis; for example, continuous cytosine-b-D-arabinofuranoside infusion reduced seizure frequency in adult rats with pilocarpine-induced status epilepticus (Jung et al., 2004), and injection of a vascular-endothelial-growth-factor-receptor-2 antagonist lowered seizure susceptibility after fluid percussion injury in rats (Neuberger et al., 2017). However, it is still unclear how the newly generated cells might also play a role in brain repair after seizures.

Larval zebrafish exhibit primary and secondary phases of neurogenesis. The primary phase, in which a few neurons are generated to coordinate a simple network and locomotor behavior, occurs until around 16 h after fertilization. After 2–3 dpf, secondary neurogenesis starts to occur to structure a brain with a more complex hierarchy (Tropepe and Sive, 2003). Unlike mammals, where adult neurogenesis predominantly occurs in the SVZ and SGZ (Rotheneichner et al., 2013), zebrafish exhibit intense adult neurogenesis in all regions of the brain, generating 6000 new cells every 30 min on average (Schmidt et al., 2013). Neurogenesis in larval and adult zebrafish also differs in its molecular forms, as adult zebrafish express different transcriptional codes for neural precursors than larvae (Adolf et al., 2006). While seizure-induced neurogenesis has not been well studied in the zebrafish, PTZ-induced seizures in adult zebrafish increased cellular proliferation and neurogenesis in the ventricular zone of the lateral part of the dorsal telencephalon, similar to the response seen in the adult mammalian brain (Duy et al., 2017). Brain injury in adult zebrafish also stimulates newborn neuron proliferation and migration toward the injured site (Kroehne et al., 2011; Kishimoto et al., 2012; Skaggs et al., 2014). These neurogenesis capabilities raise the possibility that the adult zebrafish brain may go through similar cellular remodeling following seizures as previously demonstrated in mammalian models, although this warrants further research. While there are no reported studies on seizure-induced neurogenesis in larval zebrafish, the findings from Duy et al. (2017) suggest that despite the fact that basal adult zebrafish neurogenesis is quite different from mammals, the seizure-induced changes may in fact be more similar than one might expect, and could and help inform about the role of neurogenesis in mammalian epileptogenesis.

## Drug Uptake in Zebrafish

Larval and adult zebrafish can absorb drugs through the skin, gills, and gut. The tank water method is commonly used for drug delivery in zebrafish, allowing researchers to easily control drug concentrations in the tank water and expose hundreds of zebrafish to the drug at the same time. The mature BBB in adult zebrafish leads to the uptake of tank water-delivered drugs into the brain in a pattern consistent with that seen after systemic drug administration in mammals. For example, drugs that penetrate the BBB in mammals, such as scopolamine, diphenhydramine, and haloperidol, show a high absorption rate in the zebrafish brain. On the other hand, scopolamine N-butyl bromide and desloratadine, which do not cross the mammalian BBB, are also excluded from the zebrafish brain after tank water administration (Fleming et al., 2013). It is also an efficient method of assessing the efficacy of ASD polytherapy as multiple drugs can be easily administered at the same time.

In order for the tank water method of drug delivery to be successful, drug compounds must be absorbed and distributed to a target area without rapid metabolism and excretion. Drugs such as primidone, which has poor water solubility (Berghmans et al., 2007), are not suitable for the tank water screening method as the dermal uptake rate of hydrophobic drug compounds is less than 50%. The importance of drug absorption studies in zebrafish has been previously highlighted (Sukardi et al., 2011). While the tank water method is convenient and high throughput, it does not indicate how much of each drug compound is taken up per fish. Drug intake levels in the adult zebrafish brain can be directly measured from a pool of three whole brains, whereas such measurements are challenging in micro-sized larval brains (Mussulini et al., 2013). Inappropriate drug doses and poor absorption can result in false negatives. Drug administration through alternative routes, including oral (Banote et al., 2013), intraperitoneal (Paudel et al., 2020), and retro-orbital (Pugach et al., 2010), can be used for alternative drug delivery in adult zebrafish. These techniques have a low risk of injury and death and a high success rate after a few training sessions. In addition, these alternative administration protocols permit the administration of water insoluble drugs that cannot be administered to larval zebrafish in the bathing medium.

## Seizure Phenotyping Paradigms

Zebrafish exhibit a robust seizure behavior phenotype. In both larval and adult zebrafish, behavioral changes after introducing a convulsant are transient and easy to evaluate both by manual scoring and by automatic tracking. Larval zebrafish can swim freely from 3-dpf, allowing researchers to use young larvae in behavioral studies. The behavior of small larvae is usually observed in 96-well plates, making them a truly high throughput system. However, larval behavior is simple and has a limited number of endpoints; the seizure behavior of larvae is generally analyzed based on velocity and distance traveled. Adult zebrafish have a wider spectrum of behavioral and physiological responses, such as rate of gill movements, spasms, and corkscrew swimming, and they are suitable for the novel tank test, which is used to assess anxiety-like behaviors (Wong et al., 2010). The higher-order behavior of adult zebrafish also allows cognitive psychological traits to be studied, including aggression, social preferences, anxiety, and learning/memory, meaning they are also a useful model for studying the cognitive effects of ASDs (Kundap et al., 2017; Choo et al., 2019).

Drug-induced seizures in zebrafish at all ages typically exhibit similar behavior, starting with erratic whirlpool-like swimming which develops to convulsive swimming, and eventually loss of posture or death (Afrikanova et al., 2013; Mussulini et al., 2013). Each non-drug induced epilepsy model can show different types of seizure behaviors. An adult post-traumatic epilepsy model shows reduced motor behavior with spontaneous myoclonic and generalized tonic-clonic seizures (Cho et al., 2020b). SCN1A mutant larvae exhibit increased swim activity with whole-body convulsions and rapid undirected movements (Baraban et al., 2013). STX1B mutant larvae, however, exhibit reduced swim behavior with myoclonic jerks (Schubert et al., 2014). This variety in seizure behavior, both related to animal age and to the cause of seizures, makes it difficult to create a standardized definition of behavioral seizures in zebrafish.

Another standard method used in zebrafish studies is recording single-channel local field potentials (LFPs). This method usually uses a glass electrode filled with 1 M NaCl to make recordings of up to 1 h from agarose-embedded larva (Zdebik et al., 2013). The adaptation of microfluidic technology in larvae electrophysiology has allowed electrophysiological signals to be measured from multiple zebrafish at the same time, boosting throughput in electrophysiology studies (Eimon et al., 2018; Cho et al., 2020a). With larger adult zebrafish, noninvasive multichannel electroencephalography (EEG) recordings from each hemisphere of the telencephalon and midbrain have been demonstrated, enabling the identification of seizure onset and propagation trends that cannot be determined in larvae (Cho et al., 2017).

Transparent larvae are widely used for fluorescent imaging. Generation of transgenic zebrafish expressing GCaMP, a DNA-encoded calcium indicator, coupled with advances in optical engineering, has enabled neuronal activity to be tracked in transgenic larvae at single-cell resolution (Turrini et al., 2017). The transgenic transparent *casper* adult zebrafish was introduced for *in vivo* fluorescent imaging in adult zebrafish (White et al., 2008). While calcium imaging in adult zebrafish epilepsy models has not yet been reported, it has been reported in other adult zebrafish studies (Huang et al., 2020). The continued refinement and use of state-of-the-art engineering techniques are providing us with novel neurophysiological tools, accelerating the use of the zebrafish as a valuable animal model in neuroscience.

## Conclusion

The zebrafish is a powerful tool with larval and adult zebrafish having distinct features that must be considered in epilepsy research. Mammalian seizures can be recapitulated in both stages molecularly, behaviorally, and electrophysiologically. Transient larval – adult duality means that both stages exhibit advantages and disadvantages when used as models for human epilepsy. Larvae allow for higher-throughput experiments than adults, but adult zebrafish have a unique set of advantages. Though larger than larvae, they are still considerably more economical than the mammalian models frequently used for studying epilepsy and have much higher throughput capacity than mammals. Their larger body size makes them easier to handle than larvae, and more adaptable to laboratory techniques such as dissection, injection, and multichannel EEG. Important sex differences can be studied in adults but not in larvae. Moreover, the full range of brain functions in adults can present significant advantages over larvae in pathological and pharmacological studies of epilepsy.

## Author Contributions

S-JC wrote the manuscript and generated the tables. EP, AB, and AYR edited the manuscript. All authors contributed to the article and approved the submitted version.

## Conflict of Interest

The authors declare that the research was conducted in the absence of any commercial or financial relationships that could be construed as a potential conflict of interest.
